# Embryo Development inside Female Salamander (*Ambystoma jeffersonianum-laterale*) Prior to Egg Laying

**DOI:** 10.1371/journal.pone.0091919

**Published:** 2014-03-20

**Authors:** Noah D. Charney, John J. Castorino, Megan J. Dobro, Sarah L. Steely

**Affiliations:** School of Natural Science, Hampshire College, Amherst, Massachusetts, United States of America; Laboratoire de Biologie du Développement de Villefranche-sur-Mer, France

## Abstract

The length of embryo retention prior to oviposition is a critical evolutionary trait. In all oviparous salamanders, which include the vast majority of species in the order, fertilization is thought to occur at the time of egg laying. Embryos then enter the first cleavage stage several hours after being deposited. This pattern holds for previously studied individuals in the *Ambystoma jeffersonianum-laterale* complex. Here, we document an instance in which a female *Ambystoma jeffersonianum-laterale* was carrying embryos internally that had already reached stage 10 of development. Development likely began several days prior to the start of migration to the breeding pond. This is the first such record for any egg-laying salamander, and suggests a degree of plasticity in the timing of fertilization and development not previously recognized. Further work is needed to ascertain the prevalence, mechanics, and evolutionary significance of this phenomenon.

## Introduction

In oviparous taxonomic groups, variation in the length of time that embryos are retained internally has been studied as an important evolutionary link between oviparity and viviparity, with potential to yield insight into the origin of the amniote egg [1]–[4]. Reproductive strategies among the three amphibian orders, Gymnophiona (caecilians), Anura (frogs), and Caudata (salamanders), include viviparity (defined here as giving birth to maternally nourished, fully metamorphosed juveniles), ovoviviparity (giving birth to un-metamorphosed larvae nourished only from yolk sacs), and oviparity (giving birth to unhatched eggs). Both viviparity and ovoviviparity require internal fertilization in order for developing embryos to be retained internally, whereas oviparity can occur with either internal or external fertilization. All caecilians have internal fertilization, with roughly equal numbers of known viviparous and oviparous species [5]. At least one oviparous caecilian is known to lay eggs in various stages of early development [4]. Of the more than 5,000 frog species, 10 are thought to have internal fertilization, and these species include oviparous, ovoviviparous and viviparous strategies [6].

**Figure 1:**
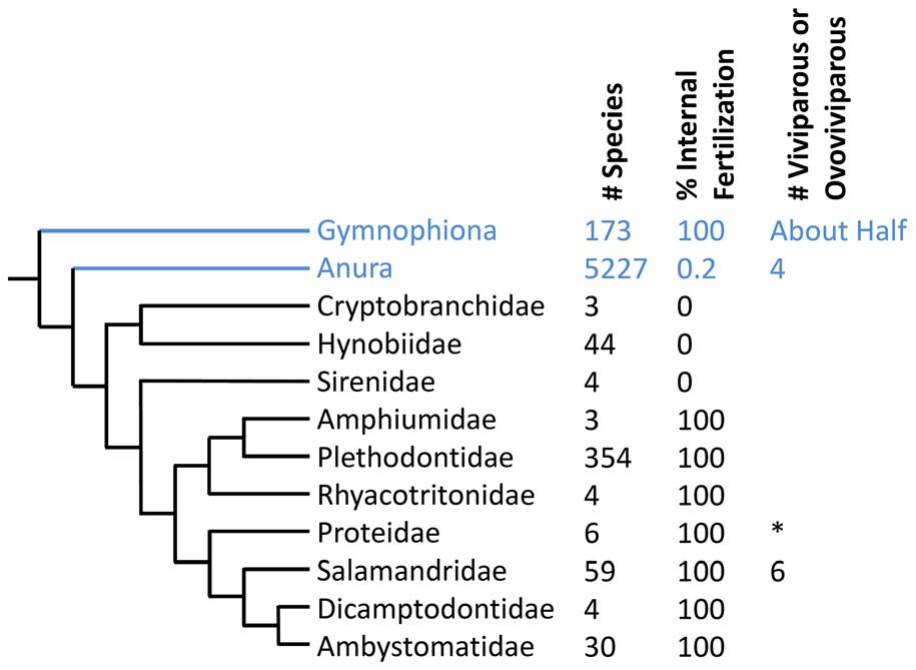
Reproductive strategies among extant amphibian species. Numbers represent estimates of the total number of species in each group, the percent of known species thought to have internal fertilization, and the number of species thought to be either viviparous or ovoviviparous. In groups with no number in the “viviparous or ovoviviparous” column, all species are oviparous. The * denotes a single oviparous species with unconfirmed accounts of it giving birth to free swimming larvae under abnormal laboratory conditions [12], [13]. Salamander families are shown in black, and the current study focuses on species in the Ambystomatidae family. Branch lengths are not meaningful [6], [9], [31]–[33].

In the vast majority of salamander species, fertilization and development is thought to always follow the same pattern of oviparity: fertilization occurs in the cloaca during the few minutes preceding oviposition [7]. Thus, while fertilization is internal, embryo development is entirely external. This understanding has held in place for well over 100 years [8], with few exceptions. Exceptions to this pattern include three primitive families of oviparous salamanders (Cryptobranchidae, Hynobiidae, Sirenidae) with completely external fertilization [9] (Figure 1). In addition, six species in two genera of Salamandridae reproduce through either ovoviviparity or viviparity, with at least one species (*Salamandra salamandra*) that can use both modes [10], [11]. Finally, there are controversial historical reports of one typically oviparous Proteid species (*Proteus anguinus*) giving birth to free swimming larvae under abnormal laboratory conditions, although this remains unconfirmed [12], [13]. Here, we present the first documented observation of fertilization and embryo development occurring substantially prior to oviposition in an egg-laying salamander.

### Study System

**Figure 2:**
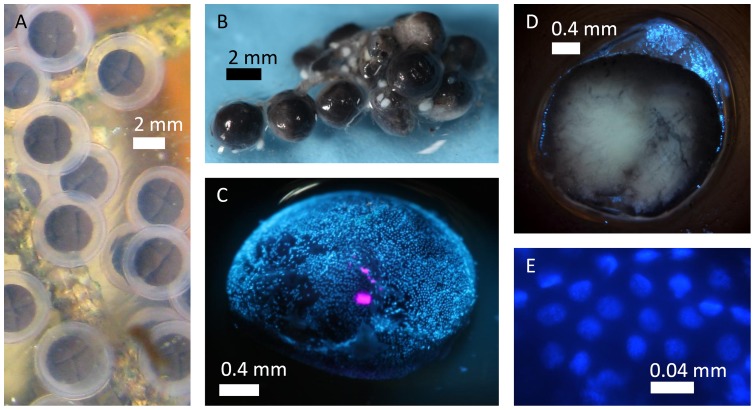
*Ambystoma jeffersonianum-laterale* embryos. A) Typical *Ambystoma jeffersonianum-laterale* eggs in cleavage stage 4 laid in a pond in western Massachusetts. B) A portion of a clutch extracted from a road-killed unisexual *Ambystoma jeffersonianum-laterale*. C) Fluorescence image of a DAPI-stained embryo from the clutch of the road-killed individual in approximately development stage 10. The blue dots are cell nuclei, the pink dots are surface reflections of the light source. D) A sectioned embryo showing a fluid-filled center with nuclei distributed around the periphery. E) A close up of nuclei in an embryo.

Members of the unisexual *Ambystoma* complex, including *A. jeffersonianum, A. laterale*, and various hybrid biotypes are thought to follow the typical life history pattern of oviparous salamanders. Adults migrate during rainy nights in the spring from upland habitat to breeding ponds. In a breeding season, a female typically mates, deposits a clutch of eggs in a single pond and then returns to the uplands all within a few weeks [14]. During mating, males place spermatophores under water, which females pick up with their cloacal lips. Studies have shown that females of some *Ambystoma* species have the ability to store sperm for an extended period of time, although how long each species can store sperm for is not fully resolved [15]–[17]. Eggs are deposited under water shortly after fertilization and enter the cleavage stage several hours after being laid (Figure 2A) [18]. In unisexual *Ambystoma*, it is thought that sperm is usually necessary only to initiate cleavage in the embryo and that sperm do not usually contribute DNA to the developing offspring. There is some evidence that paternal DNA is occasionally incorporated into the developing offspring, and no evidence that development can proceed in the absence of sperm [19]–[20].

## Materials and Methods

On the evening of March 31, 2013, we collected a dead female *Ambystoma jeffersonianum-laterale* from a rural highway in Montague Town, Franklin County, Massachusetts, USA. This is a location where we regularly witness annual migrations of salamanders crossing between upland habitat west of the road to a breeding pond east of the road. The surrounding landscape is composed of a mix of fields, forests, and houses on both sides of the road. West of the road, the land is primarily forested, with the nearest known pond approximately 250 m west. East of the road, there are two ponds within 100 m of the roadkill location in a landscape mostly covered by fields. Based on external morphology and previous work in this area, members of this population likely consist of diploid *Ambystoma jeffersonianum* individuals and triploid LJJ members of the unisexual lineage [21]. On this occasion, other live salamanders were crossing east towards the pond, and there were other dead salamanders as well. No animals were killed for this study and no live animals were used in this study. The salamander we collected had been hit by a car, and her eggs were partially spilling out of her ruptured side onto the pavement, although still firmly attached. We placed the salamander in a −20°C freezer within 30 minutes of collection, and later transferred the specimen to methanol until dissections could be performed. We are in the process of archiving our samples with the Department of Herpetology at the American Museum of Natural History (New York, NY, USA).

We extracted DNA from the salamander skin and sequenced a portion of the mitochondrial D-loop approximately 470 bp long, following Charney et al. [21]. We compared our sequence results to available sequences on GenBank using the BLAST tool. We dissected the eggs from the abdomen and transferred them to a 4% paraformaldehyde solution in phosphate buffered saline (PBS; Figure 2B). We inspected 8 of approximately 72 eggs using fluorescent microscopy. We sectioned eggs by transferring them to a 30% sucrose solution for 24 h, then freezing at −80°C for 24 h, and then cutting in half. Finally, we mounted the samples in anti-fade reagent containing DAPI [22]. We examined the eggs in an Olympus BH2 fluorescent microscope using the microscope's UV light source and a Canon Rebel T4i DSLR camera to capture images.

## Results

Mitochondrial sequences matched 100% to known sequences from the *Ambystoma* unisexual lineage on GenBank, which differ from both of the other candidate local species (*A. jeffersonianum* and *A. laterale*) by approximately 60 bp over the sequenced region [21]. The embryos collected from the road-killed salamander that were stained with DAPI each contained several hundred nuclei. Because DAPI binds to DNA, these nuclei fluoresce under UV light (Figure 2C). In sectioning the embryos, we observed that the nuclei were concentrated in a thin layer in the outer part of the embryo surrounding a fluid-filled cavity (Figure 2D), consistent with the morphology of a blastoderm surrounding a blastocoel. Each nucleus was approximately 0.02 mm in diameter and irregularly ellipsoidal in shape (Figure 2E). The embryos appeared to be in approximately stage 10 (late blastula – early gastrula) of development [18], [23].

## Discussion

These observations of early fertilization and embryo retention in an egg-laying salamander expand our understanding of plasticity in salamander life histories. Developmental plasticity is well documented for other amphibian stages, including facultative paedomorphosis in five salamander families [24], altered timing of egg hatching in response to environmental cues [25], morphological differences among larvae in the presence of predators or competitors [26], and variation in the size at metamorphosis [27]. Amphibian biologists may not be surprised to learn of further plasticity in amphibian reproduction. Perhaps the surprise is that the phenomenon we report has not previously been documented, especially given that our focal genus, *Ambystoma*, has been extremely well studied.

Typical *Ambystoma* eggs are fertilized at the time of oviposition, and laid as single undivided cells [18], yet we have shown that fertilization and development can begin long before oviposition. In contrast, other egg masses from the *Ambystoma jeffersonianum-laterale* complex in Massachusetts show the standard sequence of development (Figure 2A). In lab experiments with *Ambystoma mexicanum*, stage 10 of development was not reached until 30–65 hours post fertilization at 18°C [23], and in *A. maculatum*, stage 10 was not reached until over 60 hours at 15°C [18]. The embryos that we observed would have been developing much more slowly, as air temperatures in the preceding three days ranged from −6°C to 13°C and the maximum temperature in the salamander's underground burrow would have been 8°C, the mean annual above-ground temperature (http://ncdc.noaa.gov) [28].

Embryo development likely began before the female started migrating towards the pond because migration typically occurs on rainy evenings, the weather had been dry prior to the evening we found the salamander, and less than 3 hours elapsed between nightfall and when we placed the salamander in the freezer. This may suggest that the eggs were activated and began cleavage in the absence of sperm, or were fertilized using sperm stored from a prior breeding season. Using stored sperm from previous years is suggested by the observations of Tennessen and Zamundio [17] in *A. maculatum*, however, laboratory work with *A. opacum* implies that their sperm can only survive 6 months [15]. The female could have mated on land, however, *A. opacum* is the only species in the genus known to mate on land. Alternatively, the female could have already visited the breeding pond or another pond, mated, and then returned to land without depositing her eggs, although such behavior would be extremely atypical [14].

The ability to initiate development prior to arrival at the breeding pond has clear adaptive significance. Amphibians that breed in vernal pools compete in a growth race after spring thaws, as the larvae that get bigger faster will be less likely to be eaten, more likely to eat others, and more likely to complete metamorphosis prior to pond desiccation. Thus, giving embryos a head-start on development is a good general strategy for these species. The ability to fertilize eggs outside of the pond using sperm stored from encounters with males in prior years should be particularly beneficial for unisexual *Ambystoma*. Males are often quite rare in this system, so females cannot always count on finding a male during a breeding season [14]. Increased reliance on stored sperm could help explain how unisexuals are able to persist in ponds with few host males.

In most salamanders, it is thought that fertilization usually occurs in the cloaca or at the end of the oviduct near where it opens into the cloaca [29], [30]. In the final days of our salamander's life, she must have carried her 72 developing embryos in her oviducts, as her cloaca would likely have been too small. If sperm was involved with initiating cleavage, fertilization could not have occurred in the cloaca. Instead, the sperm would have had to travel at least a short distance up the oviducts to reach the eggs. This represents a significant departure from the idea that eggs are fertilized as they pass through the cloaca during oviposition.

While this is an isolated observation, it demonstrates the capacity for at least at least *A. jeffersonianum-laterale* to initiate development well before oviposition. Further work is needed to determine the prevalence, anatomical mechanics, life history trade-offs, and evolutionary context of embryo retention, both within this complex, and among other species. We recommend that future researchers take advantage of road-killed individuals for such study to limit the impact on these species of conservation concern.
